# SENP3 promotes tumor progression and is a novel prognostic biomarker in triple-negative breast cancer

**DOI:** 10.3389/fonc.2022.972969

**Published:** 2023-01-09

**Authors:** Youzhi Zhu, Jiasheng Zhang, Liangfei Yu, Sunwang Xu, Ling Chen, Kunlin Wu, Lingjun Kong, Wei Lin, Jiajie Xue, Qingshui Wang, Yao Lin, Xiangjin Chen

**Affiliations:** ^1^ Department of Thyroid and Breast Surgery, The First Affiliated Hospital of Fujian Medical University, Fuzhou, China; ^2^ Department of Breast Surgery, the First Hospital of Fuzhou, Fuzhou, China; ^3^ Key Laboratory of OptoElectronic Science and Technology for Medicine of Ministry of Education, College of Life Sciences, Fujian Normal University, Fuzhou, China; ^4^ Central Laboratory at The Second Affiliated Hospital of Fujian Traditional Chinese Medical University, Innovation and Transformation Center, Fujian University of Traditional Chinese Medicine, Fuzhou, China

**Keywords:** TNBC, SENP family, SENP3, WGCNA, prognosis

## Abstract

**Background:**

The clinical outcome of triple-negative breast cancer (TNBC) is poor. Finding more targets for the treatment of TNBC is an urgent need. SENPs are SUMO-specific proteins that play an important role in SUMO modification. Among several tumor types, SENPs have been identified as relevant biomarkers for progression and prognosis. The role of SENPs in TNBC is not yet clear.

**Methods:**

The expression and prognosis of SENPs in TNBC were analyzed by TCGA and GEO data. SENP3 coexpression regulatory networks were determined by weighted gene coexpression network analysis (WGCNA). Least absolute shrinkage and selection operator (LASSO) and Cox univariate analyses were used to develop a risk signature based on genes associated with SENP3. A time-dependent receiver operating characteristic (ROC) analysis was employed to evaluate a risk signature’s predictive accuracy and sensitivity. Moreover, a nomogram was constructed to facilitate clinical application.

**Results:**

The prognostic and expression effects of SENP family genes were validated using the TCGA and GEO databases. SENP3 was found to be the only gene in the SENP family that was highly expressed and associated with an unfavorable prognosis in TNBC patients. Cell functional experiments showed that knockdown of SENP3 leads to growth, invasion, and migration inhibition of TNBC cells *in vitro.* By using WGCNA, 273 SENP3-related genes were identified. Finally, 11 SENP3-related genes were obtained from Cox univariate analysis and LASSO regression. Based on this, a prognostic risk prediction model was established. The risk signature of SENP3-related genes was verified as an independent prognostic marker for TNBC patients.

**Conclusion:**

Among SENP family genes, we found that SENP3 was overexpressed in TNBC and associated with a worse prognosis. SENP3 knockdown can inhibit tumor proliferation, invasion, and migration. In TNBC patients, a risk signature based on the expression of 11 SENP3-related genes may improve prognosis prediction. The established risk markers may be promising prognostic biomarkers that can guide the individualized treatment of TNBC patients.

## Introduction

Worldwide, breast cancer has the highest incidence rate among women. Its incidence and mortality are expected to increase evidently in the coming years (1). The new definition of breast cancer molecular subtypes includes HER2 overexpression, triple-negative breast cancer (TNBC), luminal A, luminal B and other special subtypes (2). Of these, TNBC is a subtype of breast cancer, accounting for 10-20% of invasive breast cancers (3). In TNBC patients, estrogen receptor (ER), progesterone receptor (PR), and HER2 are not expressed (4). Due to the lack of PR, ER, and HER2 expression, it is not only insensitive to endocrine therapy but also insensitive to HER2 targeted therapy, and there is still a lack of FDA-approved targeted therapy drugs. Therefore, the main treatment strategies for TNBC are still surgery and chemotherapy. Although research on the related treatment of TNBC has been carried out with good results (5), the clinical outcome of TNBC is still wretched. Thus, it is still imperative to search for new biomarkers for TNBC diagnosis and prognosis and effective therapeutic targets.

The small ubiquitin-associated modifier (SUMO) family proteins are ubiquitin protein families discovered more than two decades ago (6). Humans have four SUMO forms (SUMO1, SUMO2, SUMO3 and SUMO4), while yeast, insects and nematodes have only one SUMO gene called Smt3 (7). Sumo-specific activating enzymes (E1), binding enzymes (E2) and ligases (E3) act on SUMO modification, which is quite different from the E1, E2 and E3 required for ubiquitin modification (8). As a type of posttranslational modification, Sumo is catalytically coupled with cellular protein substrates, which have the function of changing cell localization, protein interaction and biological function. It is a key regulatory link in many signaling pathways (9). SUMOylation is dynamic and reversible, and the proteins of the SENPs (SUMO specific proteases) family will quickly release the binding of SUMO acylated proteins (10), which is called deSUMOylation. Six human SENPs exist, SENP1, SENP2, SENP3, SENP5, SENP6, and SENP7, and SENPs consist of three subfamilies, each with specific cellular localization and substrate preference (11). SENP1 and SENP2 belong to the same subfamily, which has extensive substrate specificity. SENP3 and SENP5 are the second subfamily, which are nucleolar proteins that specifically bind to SUMO2/3. SENP6 and SENP7 are the last subfamily. There are nucleocytoplasmic proteins with an obvious proteolytic tendency to SUMO2 and SUMO3 (12). SENPs support normal physiological functions by maintaining the equilibrium between SUMOylation and deSUMOylation of target proteins (13). Several studies have investigated the role of SENP isotypes in various diseases, including thyroid cancer, colon cancer, atherosclerosis, prostate cancer, and pancreatic cancer (14). In several cancers, SENP overexpression can be used as a prognostic marker (15) Mechanistic studies have revealed that silencing SENPs can interfere with cancer progression and metastasis (16–19). In addition to slowing tumor growth, SENP silencing can make cells sensitive to anticancer therapy. Radiosensitization of hepatoma cells was induced by knockdown of SENP6 (20). In conclusion, these data point to the regulatory role of SENPs in cancer invasion, progression and metastasis. However, until now, the function of SENPs in TNBC has been unclear. It remains to be seen whether SENPs are useful therapeutic targets for TNBC and what their biological function is.

This study strived to systematically and comprehensively explore the expression of the SENP family in TNBC and to clarify its potential role in TNBC. We systematically incorporated the expression profiles of TNBC and analyzed the possible regulatory mechanisms through bioinformatics analysis. At the same time, based on SENP3-related genes, individualized prognostic characteristics of TNBC patients were established. This study lays the foundation for further in-depth research on the individualized treatment of TNBC.

## Materials and methods

### Extracting SENP expression and patient clinical data from TNBC datasets

The Gene Expression Omnibus (GEO) database (http://www.ncbi.nlm.nih.gov/geo/) and The Cancer Genome Atlas (TCGA) database (http://www.cbioportal.org/) were used to extract the mRNA expression levels of SENPs and the clinical profiles of TNBC. GSE45827, GSE53752, GSE65216, and GSE58812 were obtained from the GEO database. TCGA consisted of 114 cases of adjacent normal breast tissue and 166 TNBC tissue. GSE45827 consisted of 11 cases of adjacent normal breast tissue and 41 TNBC tissue. GSE53752 consisted of 25 cases of adjacent normal breast tissue and 51 TNBC tissue. GSE65216 consisted of 11 cases of adjacent normal breast tissue and 55 TNBC tissue. GSE58812 had 107 TNBC samples with complete survival information. The data extraction method is based on our previous study (21).

### DNA constructs

The sequences for SENP3-shRNA1 (5’-CATTGGTCCCTCATCTCTGTT-3’) and SENP3-shRNA2 (5’-CCTCGCTGACATTCCACTGGA-3’) were cloned into the PLVX vector.

### Cell culture and cell transfection

Breast cancer cells (BT-549 and MDA-MB-231) were obtained from American Type Culture Collection (ATCC, Manassas, VA, USA). BT-549 and MDA-MB-231 cells were cultivated in DMEM (Biological Industries (BI), Israel) and L-15 (BI) medium, respectively, under 5% CO_2_ at 37°C. The sequence for SENP3-shRNA was cloned into the pLVX vector. The transfection method was performed according to the transfection reagent instructions, Lipofectamine 2000 (Invitrogen, Carlsbad, CA, USA).

### RNA isolation and RT–qPCR

In this study, RNA extraction from cells was performed with TRIzol (Invitrogen, CA, USA). Reverse transcription of RNA was performed using an RNA reverse transcription kit (Takara, Japan). A SYBR Green Kit (Vazyme, China) was employed to perform RT–qPCR. Using GAPDH as an internal control, the mRNA of the senp3 gene was detected by RT–qPCR. All primers were from Sang Ya (Fuzhou, China), and the relative mRNA level was determined by the 2^- ΔΔ^ CT method.

### Proliferation assay

A 96-well plate was removed every 24 h, 10 μL of CCK (TransGen) reagent was added to each well, and the OD was measured after 1.5 hours of incubation in the original culture conditions. The OD value of all wells at 450 nm was detected, and the data were collected for analysis. The experiment was conducted three times.

### Cell migration assays

A total of 300 μL of complete medium was added to each well in the 24-well plate, and then the Transwell chamber (Falcon) was placed into the 24-well plate. A 0.5 mL cell dilution (cell density: 1 x 10^4^ cells/ml) was added to each chamber with 3 replicates in each group. After 24 h, the medium was discarded, and the cells in the upper chamber were removed. After the cells were fixed, the treated Transwell chamber was stained with gentian violet dye solution for approximately 8 min. The staining solution was discarded, and a cotton swab was used to remove dye on and around the basement membrane. Transwell chambers were photographed with a microscope.

### Invasion assays

The 24-well plate was filled with complete medium (300 mL), and a Transwell chamber filled with Matrigel (Corning, New York, USA) was added to the plate. A total of 0.5 mL of cell dilution (cell density: 1 x 10^4^ cells/ml) was added to each chamber with 3 replicates in each group. After 24 h, the medium was discarded, and the cells in the upper chamber were removed. After the cells were fixed, the treated Transwell chamber was stained with gentian violet dye solution for approximately 8 min. The staining solution was discarded, and a cotton swab was used to remove dye on and around the basement membrane. Transwell chambers were photographed with a microscope.

### Weighted gene correlation network analysis of SENP3

WGCNA is a method for analyzing gene expression patterns of multiple samples and was performed with the R software package. It assumes that the coexpression gene network obeys a scaleless distribution first and then specifies the adjacency function of the gene coexpression correlation matrix and the adjacency function constructed by the gene network, computes the difference coefficient of different nodes, and then constructs a hierarchical clustering tree.

### Functional enrichment analysis

Kyoto Encyclopedia of Genes and Genomes (KEGG) pathway analysis was performed using the functional enrichment tool DAVID4. Using the DAVID bioinformatics resources, a combined biological database and tools can be found for analysis to systematically explore the biological significance of gene sets. According to the enrichment scores, the default parameters in the tool were used to rank enriched pathways.

### Construction of the SENP3 gene risk prediction model

Based on survival in R and the glmnet software package, the genetic risk prediction model related to SENP3 was created by utilizing the least absolute shrinkage and selection operator (LASSO). Lasso is suitable for model picking of high-dimensional data that can choose prognostic correlation genes of TNBC by reducing regression coefficients. In a 10-fold cross-validation process, the optimal penalty weight for the LASSO model was discovered using a grid hunt technique. In the end, most gene pair coefficients remained zero, and only a few gene pairs with coefficients other than zero were found to be closely associated with the prognosis of TNBC.

### Statistical analysis

Statistical correlations were calculated by t tests. In comparisons of survival curves, the Kaplan–Meier method was performed, the log-rank test was used to assess survival differences with the best cutoff value. *p*<0.05 was identified as statistically significant.

## Results

### High expression of SENP3 is associated with poor prognosis in TNBC patients

**Figure 1:**
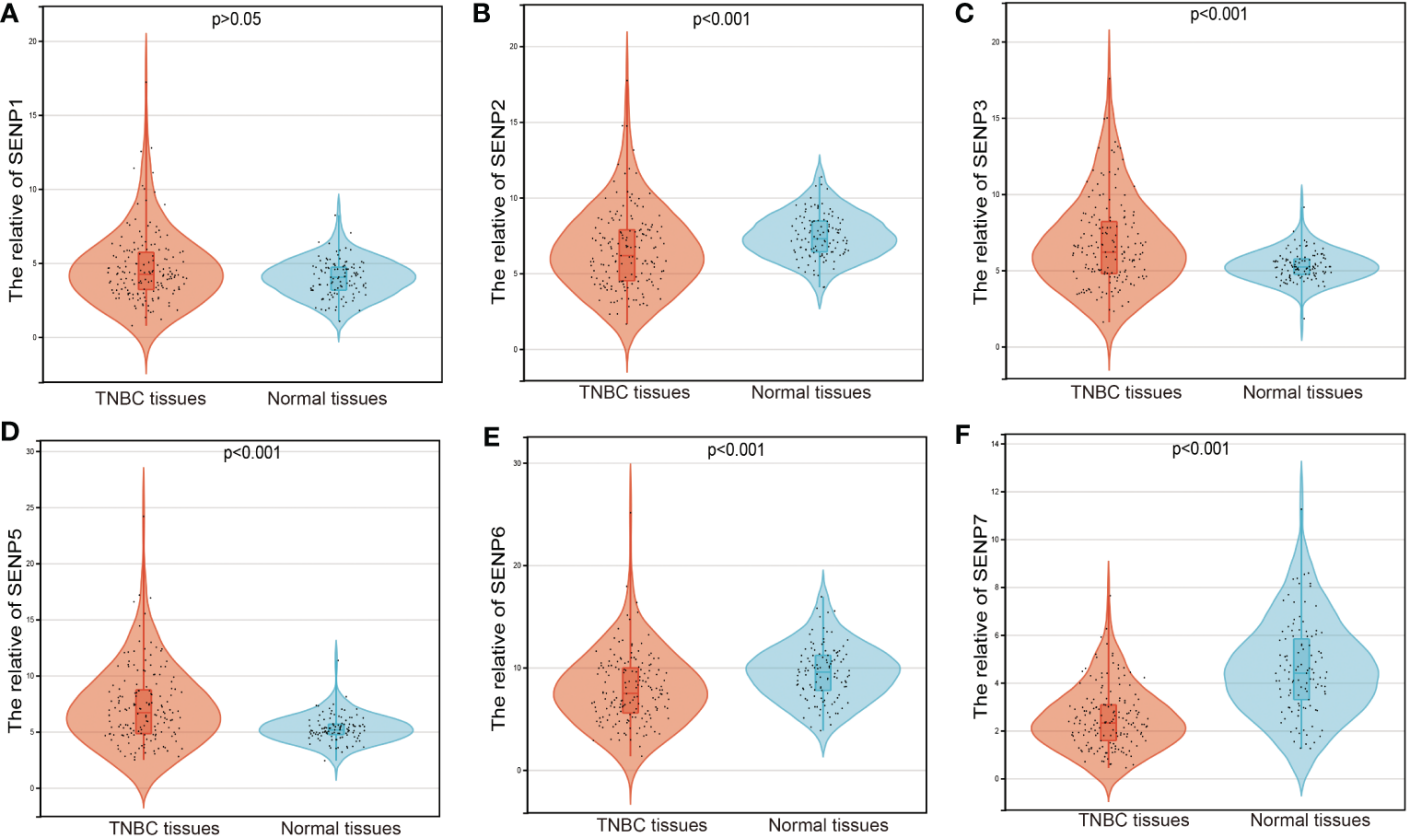
mRNA expression of SENPs in TNBC based on the TCGA database. **(A–F)** The expression of SENP1 **(A)**, SENP2 **(B)**, SENP3 **(C)**, SENP5 **(D)**, SENP6 **(E)**, and SENP7 **(F)** in TNBC.

We used an expression analysis of the SENP family in TNBC patients based on the TCGA database (**Figure 1**). The results indicated that compared with normal tissues, two genes (SENP3 and SENP5) were upregulated, three genes (SENP2, SENP6 and SENP7) were downregulated, and the expression levels of SENP1 remained unchanged in TNBC tissues.

**Figure 2:**
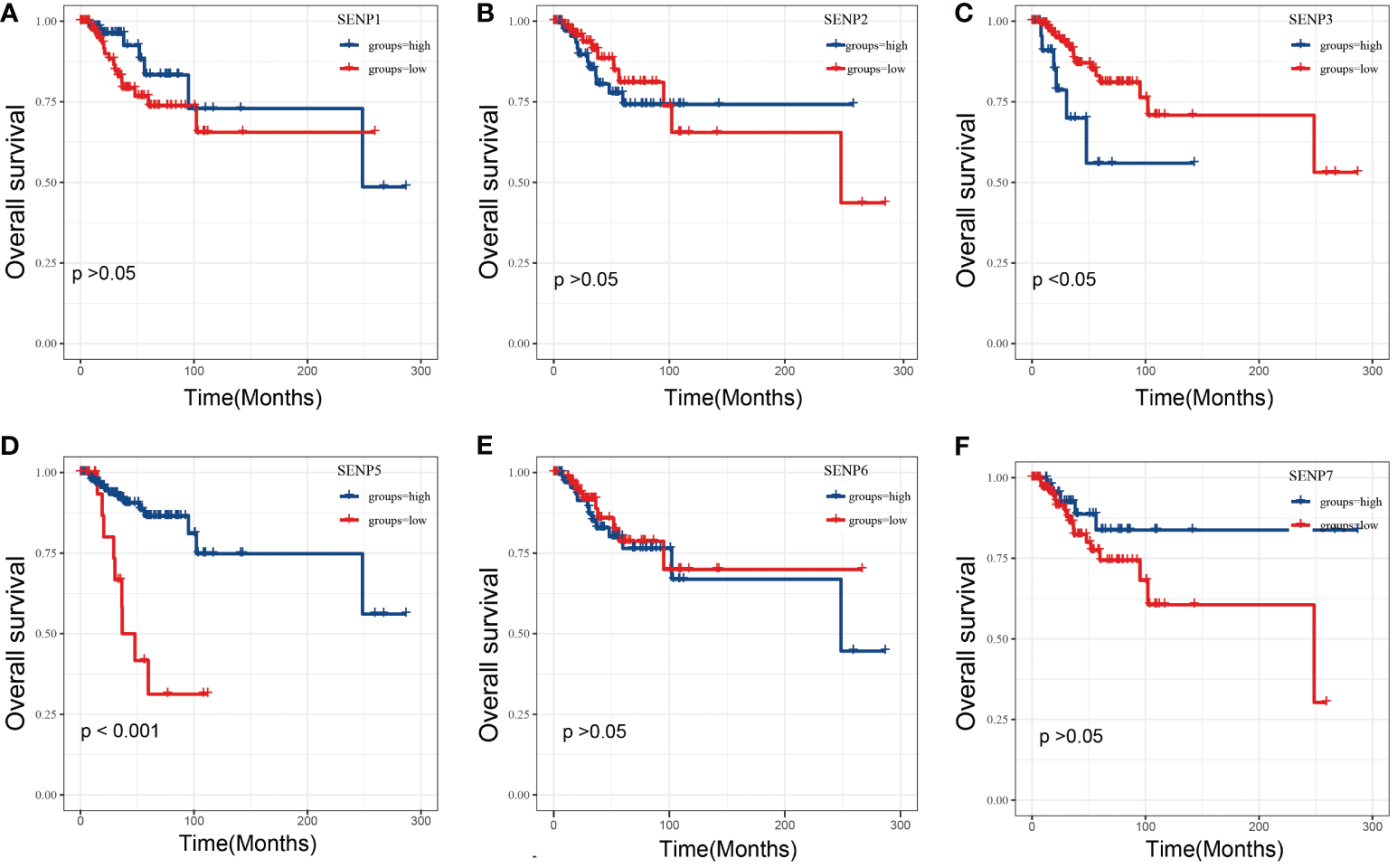
OS analysis of SENPs in TNBC patients based on the TCGA database. **(A–F)** Overall analysis of the prognostic value of SENP1 **(A)**, SENP2 **(B)**, SENP3 **(C)**, SENP5 **(D)**, SENP6 **(E)**, and SENP7 **(F)** expression for OS in TNBC patients by Kaplan–Meier analysis based on TCGA. The Kaplan–Meier method was used to draw survival curves.

To estimate the prognostic significance of the SENP family in TNBC patients, we examined the correlation between the expression of SENP family members and overall survival (OS) (**Figure 2**). High mRNA expression of SENP3 and low mRNA expression of SENP5 indicated a significantly poorer prognosis for TNBC patients (**Figures 2C, D**). However, no apparent correlations between the mRNA expression levels of SENP1, SENP3, SENP6 and SENP7 and the prognosis of patients with TNBC were found (**Figures 2A, B, E, F**).

**Figure 3:**
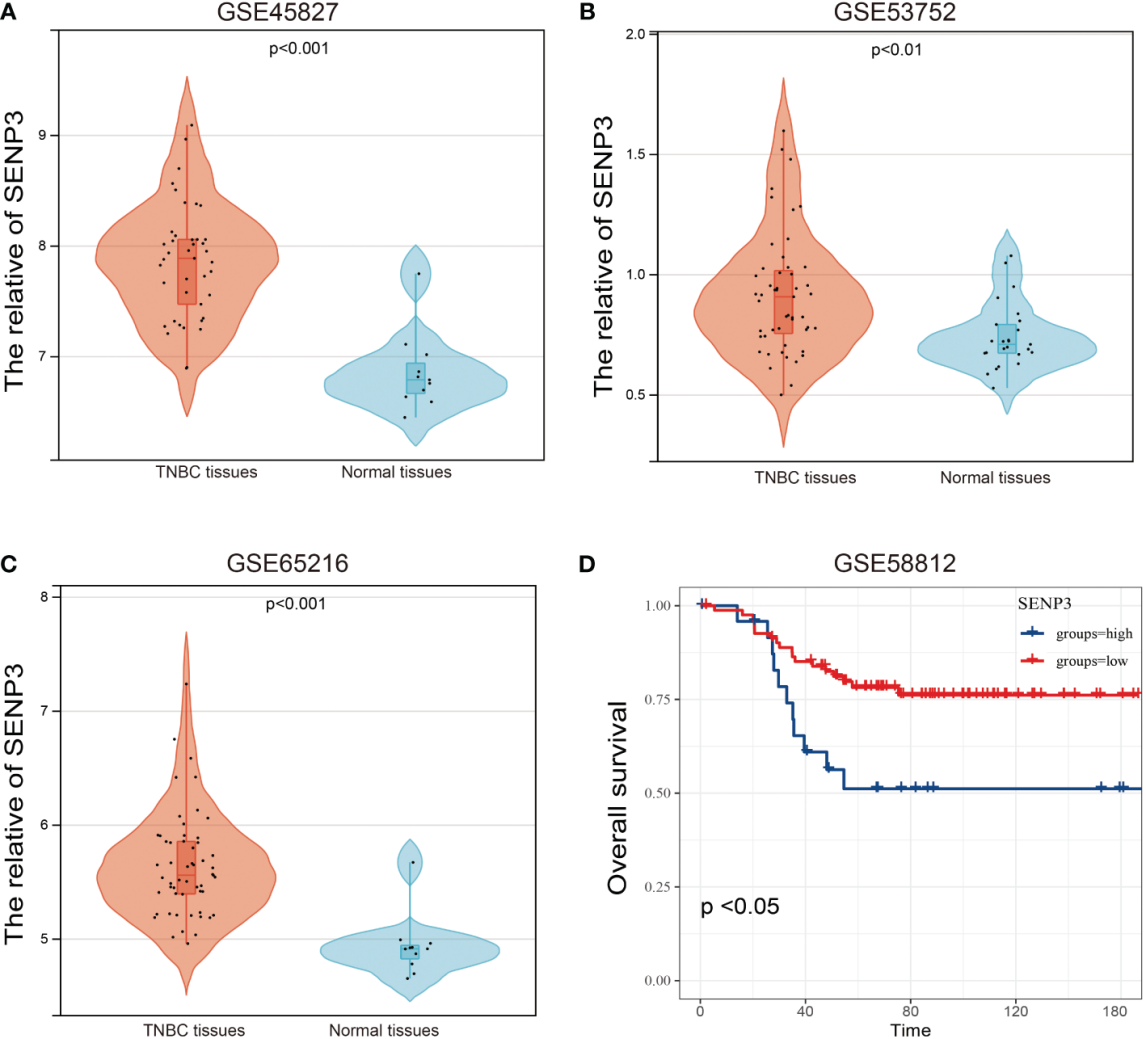
mRNA expression and OS analysis of SENP3 in TNBC based on the GEO database. The mRNA expression of SENP3 in TNBC patients in GSE45827 **(A)**, GSE53752 **(B)**, and GSE65216 **(C)**. **(D)** OS analysis of SENP3 in TNBC patients by Kaplan-Meier analysis based on GSE58812. The Kaplan–Meier method was used to draw survival curves.

To characterize the association between SENP3 expression and prognosis in TNBC, four GEO datasets, GSE45827, GSE53752, GSE65216 and GSE58812, were used. The expression of SENP3 was also significantly downregulated in normal tissues compared with TNBC tissues based on the GSE45827, GSE53752 and GSE65216 datasets (**Figures 3A–C**). Exploration in the GSE58812 cohort showed a similar outcome to that obtained from the TCGA database, high expression of SENP3 was strongly related to shorter OS in TNBC patients (**Figure 3D**). In addition, we measured SENP3 expression in other subtypes of breast cancer based on TCGA and GEO datebases. The results showed that SENP3 expression was increased in TNBC compared wtih normal tissues. However, in HER2 and Lumina subtypes of breast cancer, SENP3 expresssion was decreased compared with normal tissues (**Supplementary Figure 1**).

### SENP3 promotes TNBC cell migration, invasion and proliferation

**Figure 4:**
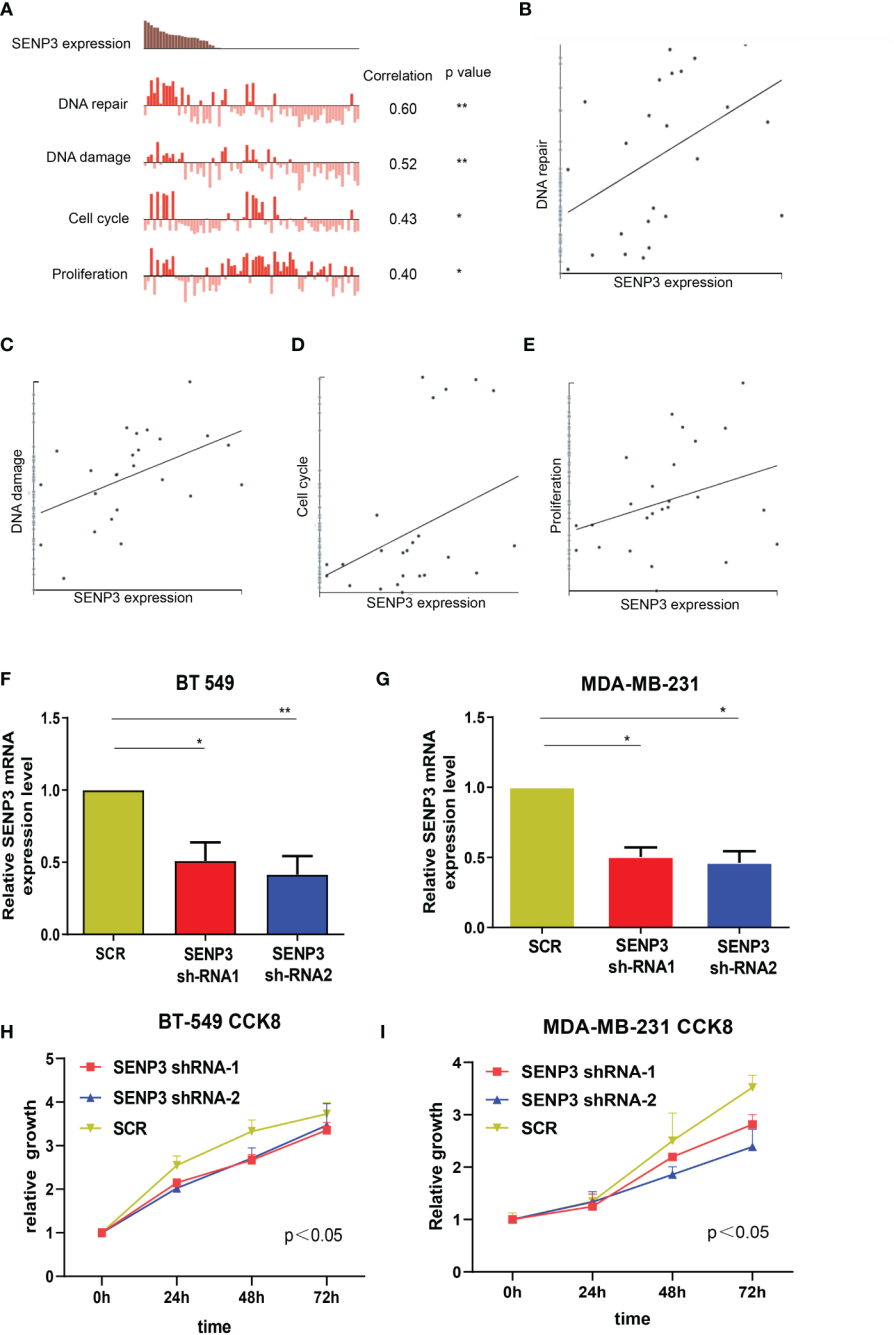
Knockdown of SNEP3 inhibits TNBC cell growth. **(A–E)** Data from Jordan NV (No. cells=70) demonstrated that SENP3 mRNA expression was positively correlated with regulation of the cell cycle, DNA damage and repair, and proliferation. **(F, G)** The SENP3 expression changes were confirmed by real-time PCR in TNBC cells (BT-549 and MDA-MB-231) after shRNA transfection. **(H, I)** The growth ability of BT-549 **(C)** and MDA-MB-231 **(E)** cells was measured by the CCK-8 assay after shRNA transfection. *, *p* < 0.05; **, *p* < 0.01.

To investigate the biological function of SENP3, CancerSEA was used for single-cell analysis. According to the data from Jordan NV (No. cells = 70), SENP3 might regulate DNA repair, DNA damage, the cell cycle and proliferation in breast cancer cells (**Figures 4A–E**). Next, we knocked down the expression of SENP3 in MDA-MB-231 cells and BT-549 cells by transfecting them with shRNA1-SENP3 and shRNA2-SENP3 plasmids. RT–qPCR analysis showed that shRNA1-SENP3 and shRNA2-SENP3 had strong inhibitory effects on SENP3 expression (**Figures 4F, G**). As a result of the CCK-8 assay, both BT-549 and MDA-MB-231 cells showed markedly decreased proliferation when SENP3 was knocked down (**Figures 4H, I**).

**Figure 5:**
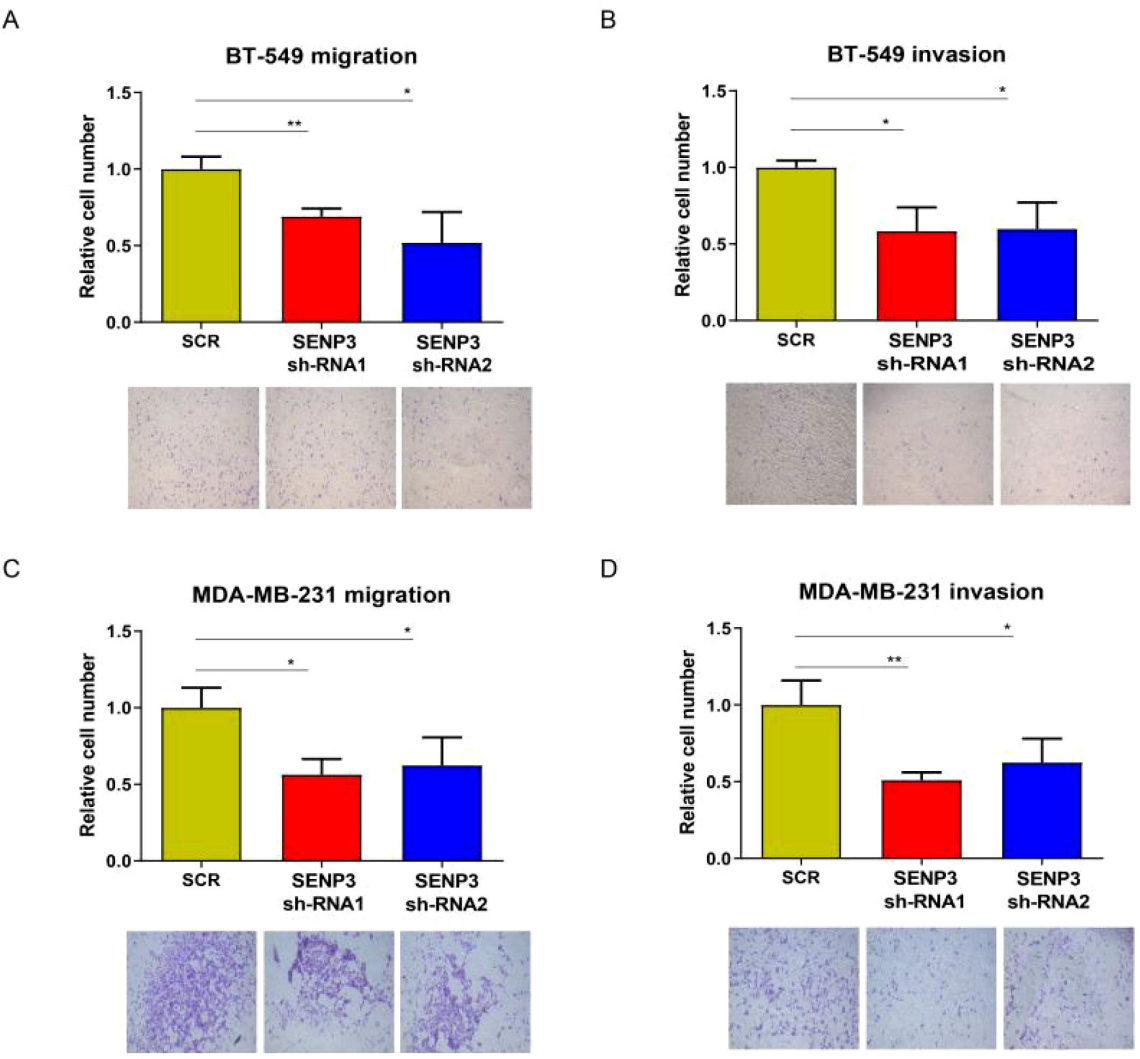
Knockdown of SNEP3 inhibits TNBC cell invasion and migration. **(A, B)** Decreased expression of SENP3 inhibited BT-549 cell migration. **(A)** and invasion. **(B)** Decreased expression of SENP3 inhibited MDA-MB-231 cell migration **(C)** and migration **(D)**. *, *p* < 0.05; **, *p* < 0.01.

Transwell analysis was used to further study whether SENP3 affects the migration and invasion of TNBC cells. The results showed that SENP3 gene knockdown inhibited the migration and invasion of BT-549 and MDA-MB-231 cells (**Figure 5**).

In conclusion, these results suggest that SENP3 knockdown suppresses the migration, invasion and proliferation of TNBC cells.

### Gene analysis of SENP3-related coexpression modules

**Figure 6:**
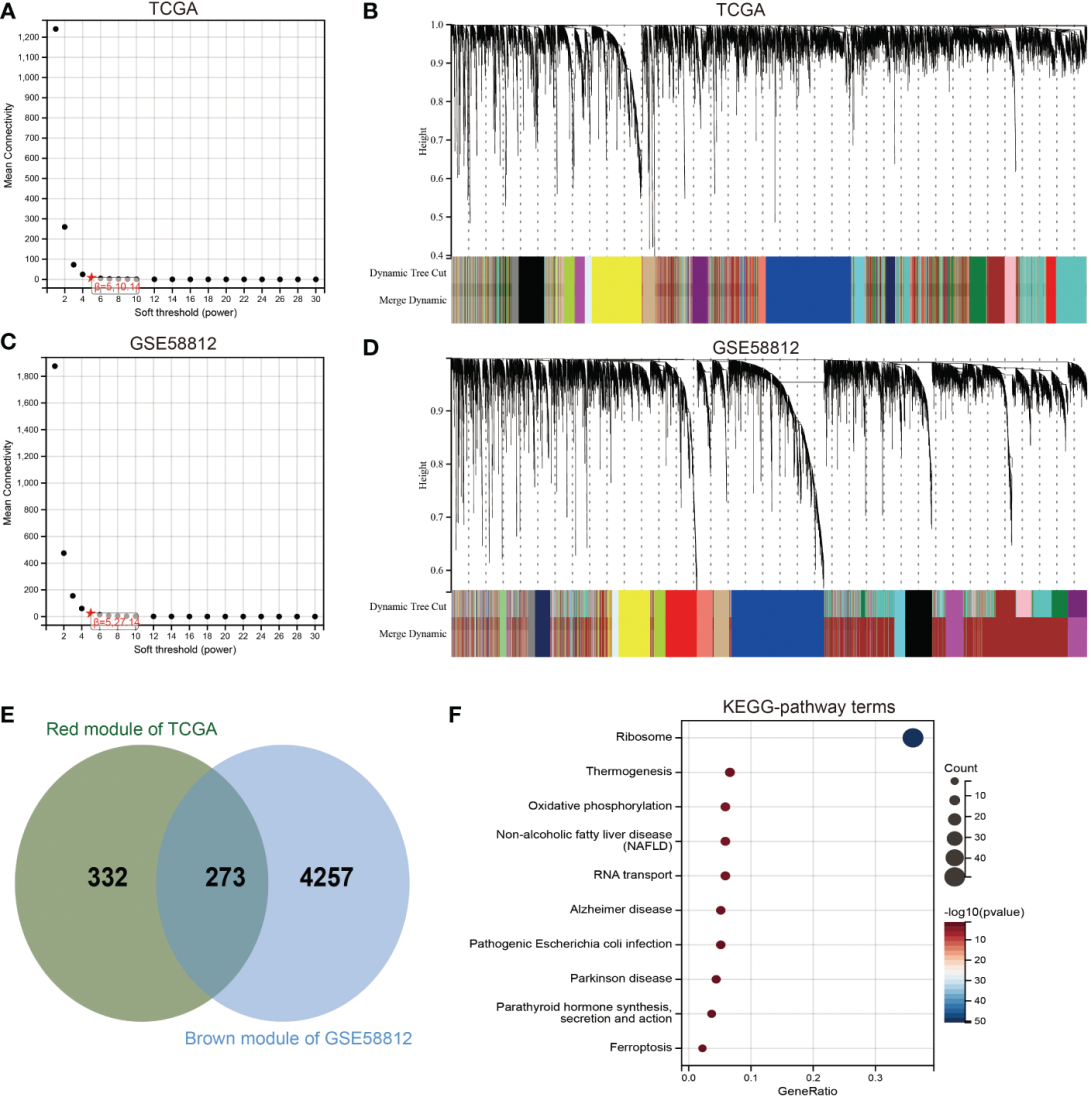
Identification of coexpression module genes associated with SENP3 using WGCNA. **(A)** Relationship between scale-free topology model fit and soft-threshold powers in the TCGA database. **(B)** Dendrogram of modules identified by WGCNA in the TCGA database. **(C)** Relationship between scale-free topology model fit and soft-threshold powers in the GSE58812 database. **(D)** Dendrogram of modules identified by WGCNA in the GSE58812 database. **(E)** The intersection of the magenta module for the GSE58812 database and the salmon module for the TCGA database contained 273 genes. **(F)** Results of KEGG analysis of 273 genes.

To gain further insights into the biological functions associated with SENP3 upregulation in TNBC, the WGCNA package was used, and the genes with highly related genes were grouped into one module. In this research, the TCGA database and GSE58812 database were used to construct a weighted gene coexpression network. To choose the best threshold power, we calculated network topologies with soft threshold powers from 1 to 30. As shown in **Figures 6A, C**, the minimum power is a power value of 5 for the scale-free topology of the GSE58812 and TCGA databases. Then, we set power=5 (GSE58812& TCGA) to generate a hierarchical clustering tree (**Figures 6B, D**). In the GSE58812 database, the red module containing 4530 genes had the highest correlation with SENP3 expression. The brown module, including 605 genes, had the highest correlation with SENP3 expression in the TCGA database. A total of 273 genes were common to the GSE58812 and TCGA datasets (**Figure 6E**). Enrichment analysis showed that the enriched biological processes of these 273 genes included ribosome, thermogenesis, oxidative phosphorylation, nonalcoholic fatty liver disease, RNA transport, Alzheimer’s disease, parathyroid hormone synthesis secretion and action, pathogenic *Escherichia coli* infection, Parkinson’s disease, and ferroptosis (**Figure 6F**).

### Construction of the SENP3-related gene prognostic model and survival analysis

**Figure 7:**
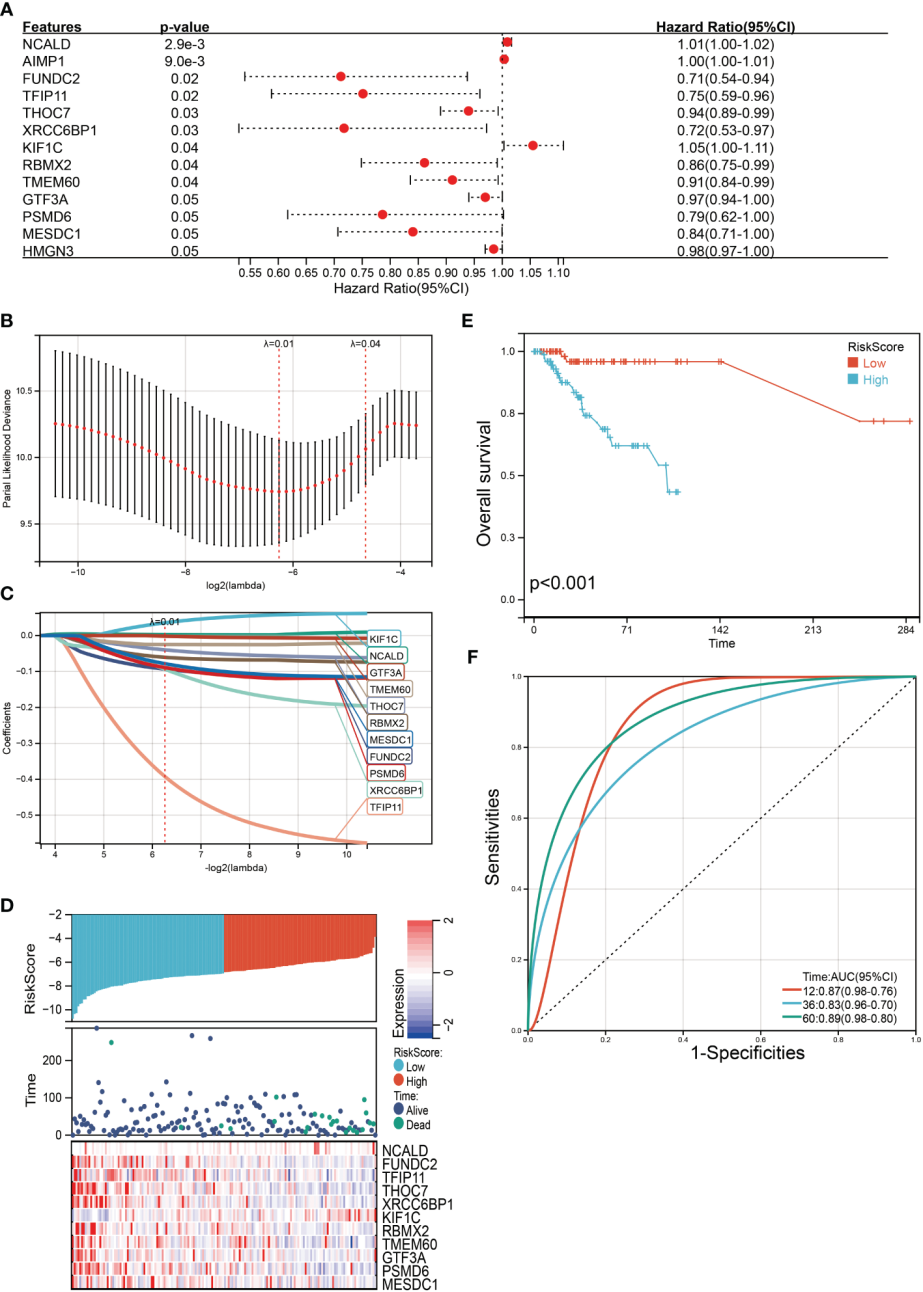
Construction of a SENP3 gene-based classifier to predict prognosis in TNBC patients. **(A)**Thirteen SENP3-related genes with statistical significance in univariate Cox analysis are highlighted with detailed outputs. **(B)** Partial likelihood deviance of OS for the LASSO coefficient profiles. **(C)** LASSO coefficient profiles of FUNDC2, TFIP11, THOC7, XRCC6BP1, RBMX2, TMEM60, GTF3A, PSMD6 and MESDC1 expression for OS. **(D)** Kaplan–Meier survival analysis showing the impact of the classifier on OS. **(E)** The distributions of the risk score, survival status, and expression of 11 SENP3-related genes in TNBC patients. **(F)** ROC curves comparing the prognostic accuracy of the classifier in TNBC patients using AUCs at 1 year, 3 years and 5 years to assess prognostic accuracy.

Next, we performed univariate Cox survival research on these 273 SENP3-related genes. Finally, the results indicated that 13 SENP3-related genes were closely correlated with the prognosis of TNBC patients. Overexpression of 10 SENP3-related genes (FUNDC2, TFIP11, THOC7, XRCC6BP1, RBMX2, TMEM60, GTF3A, PSMD6, MESDC1 and HMGN3) was weakly correlated with better OS in TNBC patients, whereas the remaining 3 SENP3-related genes (NCALD, AIMP1 and KIF1C) were prominently correlated with shorter OS in TNBC patients (**Figure 7A**). Multiple genes can be used to better predict patient prognosis. Thus, we ran the LASSO Cox regression model and calculated the regression coefficients based on the aforementioned 13 prognostic SENP3-related genes. The risk score model achieved the best fit when 11 of the 13 SENP3-related genes were included (**Figure 7B**). Risk score= (0.0356 * KIF1C) + (0.0027 * NCALD) + (-0.0932 * FUNDC2) + (-0.3926 * TFIP11) + (-0.0397 * THOC7) + (-0.0938 * XRCC6BP1) + (-0.0891 * PSMD6) + (-0.0605 * RBMX2) + (-0.0248 * TMEM60) + (-0.0015 * GTF3A) + (-0.0782 * MESDC1) (**Figure 7C**). A risk score was assigned to each TNBC patient using the formula. TNBC patients were divided into high-risk and low-risk scoring groups. The risk score, survival status, and expression of the 11 SENP3-related genes in TNBC patients are shown in **Figure 7D**. Kaplan–Meier curve analysis showed that the OS of the high-risk score group was obviously lower than that of the low-risk score group (**Figure 7E**).

**Figure 8:**
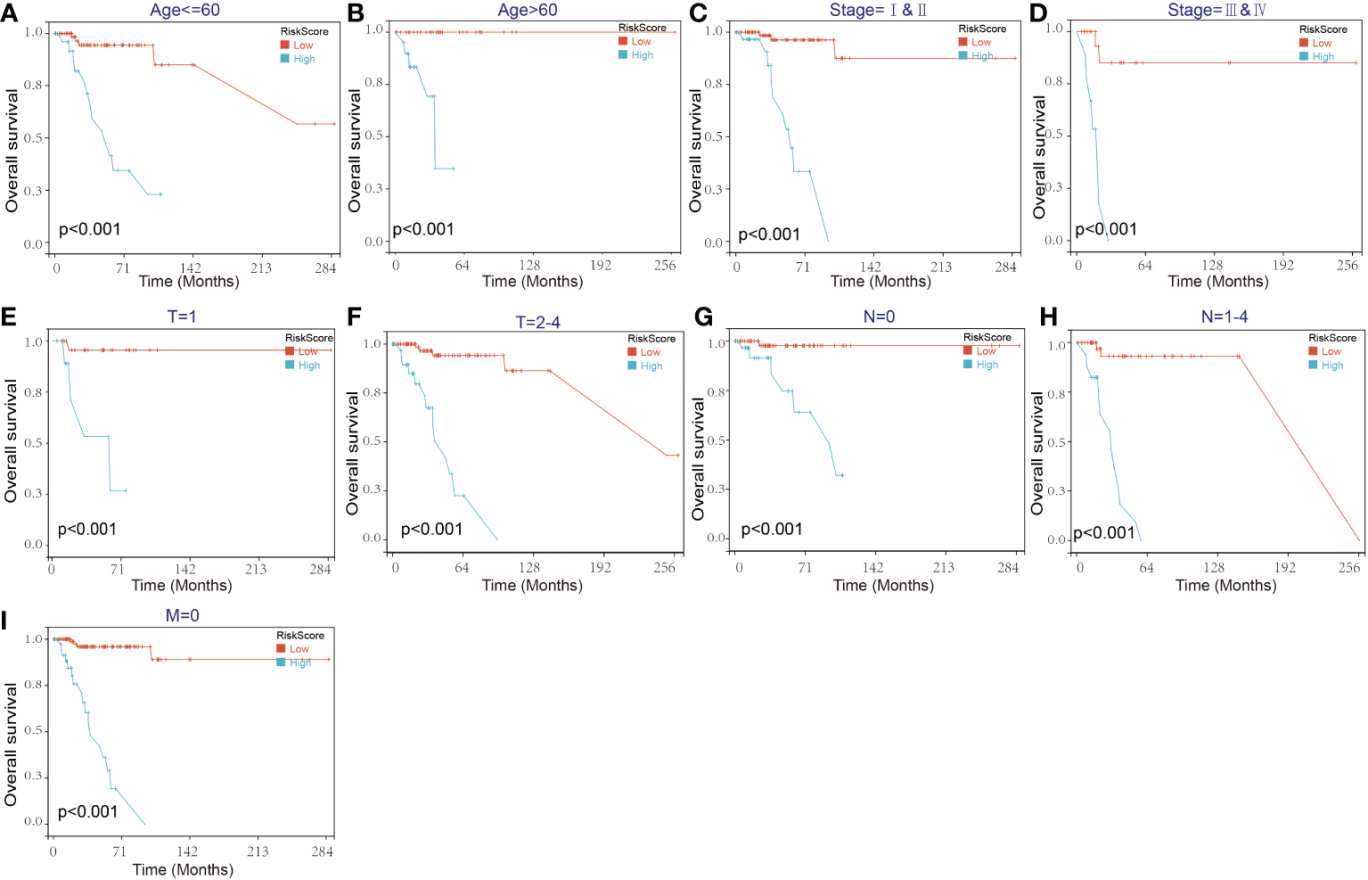
Kaplan–Meier survival curves of the OS of TNBC patients according to the risk score model in different subgroups. **(A, B)** Prognostic analysis of TNBC patients in the age<=60 **(A)** and age>60 **(B)** subgroups. **(C, D)** Prognostic analysis of TNBC patients in the stage=I&II **(C)** and stage=III& IV **(D)** subgroups. **(E, F)** Prognostic analysis of TNBC patients in the T=1 **(E)** and T=2-4 **(F)** subgroups. **(G, H)** Prognostic analysis of TNBC patients in the N=0 **(G)** and N=1-4 **(H)** subgroups. Prognostic analysis of TNBC patients in the M=0 **(I)** subgroup. The Kaplan–Meier method was used to draw survival curves.

Then, the reliability of the model was verified by the receiver operating characteristic (ROC) curve, which indicated that the area under the curve (AUC) values for 12-, 36-, and 60-month OS were 0.87, 0.83, and 0.89, respectively (**Figure 7F**). The above results show that the model has predictability value for the prognosis of TNBC patients. To further assess the robustness of risk score model, we stratified the TNBC population based on age, gender, stage and TMN. After stratification of age<=60 (**Figure 8A**), age>60 (**Figure 8B**), stage= I&II (**Figure 8C**), stage=III&IV (**Figure 8D**), stage T=1 (**Figure 8E**), stage T=2-4 (**Figure 8F**), stage N=0 (**Figure 8G**), stage N=1&4 (**Figure 8H**) and stage M=0 (**Figure 8I**), respectively, the risk score based on SENP3-related genes signature was an independent prognostic indicator, and patients with high risk scores had a poorer prognosis.

To further verify the validity and stability of the prognostic model, GSE53752 was used as the validation dataset. Each patient was brought into the previous prognostic model to calculate the risk score. Patients were divided into high-risk and low-risk groups. Kaplan-Meier curve analysis showed that TNBC patients with low-risk scores had a better OS than those in the high-risk-score group (**Supplementary Figure 2**). These results further confirmed the relatively good stratification ability of the prognostic model.

### Construction of prediction model for clinical prognosis

**Figure 9:**
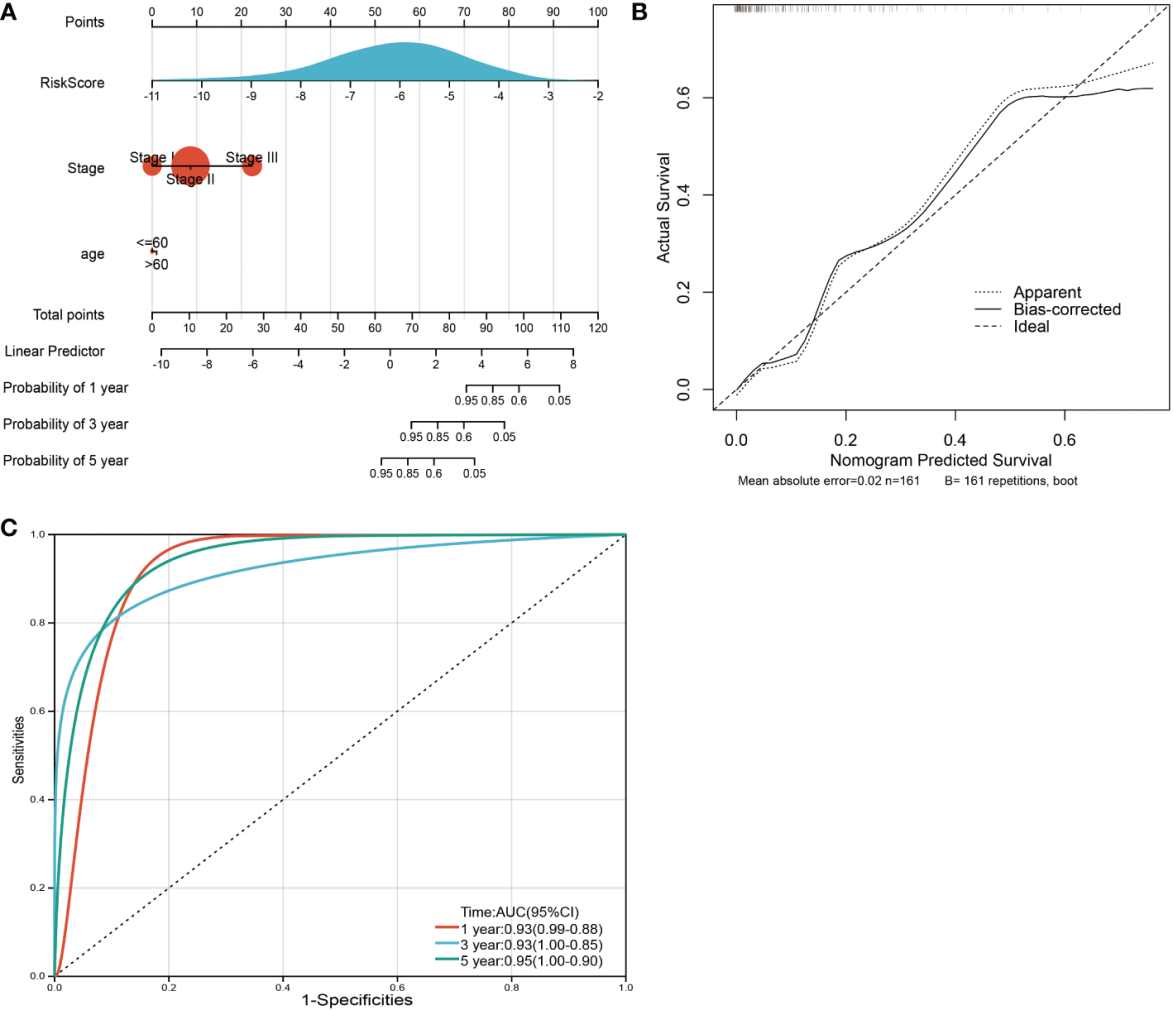
Nomogram and calibration plots for the prediction of outcomes in TNBC patients based on the risk score. **(A)** Nomogram combining age, stage and the risk score for predicting 1-year, 3-year and 5-year events. The line segment corresponding to each variable is marked with a scale, which represents the value range of the variable, and the length of the line segment reflects the contribution of the factor to the outcome event. The point in the figure represents the individual score corresponding to each variable under different values and the total score of the corresponding individual scores after all variables are taken. **(B)** The calibration plots for predicting OS. **(C)** ROC curves comparing the prognostic accuracy of the classifier in TNBC patients using AUCs at 1 year, 3 years and 5 years to assess prognostic accuracy.

To facilitate the application of our model in clinical prognosis, we integrated the risk score and established a nomogram based on 3 variable factors, including age, tumor stage, and risk model score. A survival nomogram plot was designed to accurately calculate the 12-, 24-, and 60-month survival probabilities of TNBC patients (**Figure 9A**). The calibration curve plot indicated that the nomogram achieved better results than the ideal model (**Figure 9B**). Furthermore, the AUCs for 12-, 36-, and 60-month OS were 0.93, 0.93, and 0.95, respectively, showing good accuracy (**Figure 9C**). These results indicate that the nomogram is able to predict the long-term survival of TNBC patients reasonably well.

## Discussion

Breast tumors are highly heterogeneous. There are great differences in clinical treatment and prognosis among different patients. TNBC is a complex subtype of malignant breast cancer that lacks the expression of ER PR and HER2. Therefore, targeted therapy is difficult (22). TNBC is generally considered to have high malignancy and poor prognosis. Because of its special type, endocrine therapy or molecular targeted therapy is not suitable. Thus, chemotherapy has become the major systematic treatment, but the effect of routine postoperative radiotherapy and chemotherapy is limited. Tumor recurrence may be the final outcome of residual metastatic lesions (23).

In the context of peptidase action, SENP3 disrupts protein SUMOylation and maintains cell SUMOylation homeostasis by uncoupling SUMOylated protein substrates (20). Disruption of SUMO dynamics triggers various pathophysiological conditions, including cancer, and overexpression and genetic variation of SENPs in malignancies have been reported (21). Indeed, SENPs regulate pathways such as neovascularization of the cell cycle and DNA repair, suggesting that abnormal function of SENPs is a driver of cancer and affects the homeostasis of SUMO-mediated signaling.

We detected the expression and prognosis of SENP family members in TNBC. We found that only SENP3 was highly expressed in TNBC, and the prognosis was poor.

SENP3, a member of the SENP family, changes protein modification by uncoupling target proteins. The role of SENP3 is essential for maintaining the equilibrium of SUMOylation and guaranteeing a standard protein role and cellular activity (24). As a 65 kDa protein, human SENP3 is composed of 574 amino acids and is involved in the processing and reconciliation of SUMO2/3 precursors. It plays an important role in particular processes (25). High expression of SENP3 enhanced cell migration in gastric cancer cells (26). SENP3 was confirmed to be related to gastric cancer metastasis *in vivo* models and tumor patient specimens (17). Cell differentiation, proliferation, and apoptosis are regulated by Sp3 (specific protein 3) (27). SUMO modification also acts on SP3 in glioma SP3 deSUMOylation catalyzed by SENP3 (28). In recent years, the research and functional analysis of SENP3 *in vivo* has been increasing. In the formation and development of various cancers, SENP3 has been found to play an essential role, such as head and neck cancer, ovarian cancer and oral squamous cell cancer (29–31). In mice, the deletion of SENP3 in macrophages promotes breast cancer progression and metastasis, according to recently published studies. Moreover, SENP3 may also promote cancer progression by regulating macrophage polarization (32), and high SENP3 levels are associated with more advanced tumor grades and metastasis as well as poor survival outcomes (33), meaning that SENP3 may play a role in tumorigenesis and progression. However, the function of SENP3 in TNBC is unclear. The biological role of SENP3 and its therapeutic target for TNBC need to be further studied. In this study, *in vitro* experiments showed that SENP3 knockdown in TNBC cells could interfere with proliferation, migration and invasion.

Next, 273 highly related genes and coexpression networks of SENP3 genes were identified by WGCNA. We created a predictive risk model for TNBC patients based on 11 SENP3-related genes (KIF1C, NCALD, GTF3A, TMEM60, THOC7, RBMX2, MESDC1, FUNDC2, PSMD6, XRCC6BP1, and TFIP11) by LASSO regression and Cox univariate analysis. It has been reported that these 11 genes may be related to tumor progression. KIF1C is a kinesin-like motor protein that mediates the resistance of mouse macrophages to anthrax lethal factor and is currently less studied in tumors (34). Involved in calcium signaling as well as G protein-coupled receptor signaling, NCALD is part of the neuronal calcium sensor family (35), and lower expression of NCALD in tumors is also considered to be associated with poor prognosis (36). GTF3A is considered to be associated with the migration of different types of cancer, and in colorectal cancer, high GTF3A and GTF3B expression seems to be associated with poor prognosis (37). The function of TMEM60 has also been studied in tumors, and elevated levels of TMEM60 lead to enhanced glioma proliferation, migration, and invasion, inhibit apoptosis and promote PI3K/Akt activation (38). THOC7 is a member of the THO complex associated with the mRNA export apparatus, and human THO suppresses transcription-related instability in repetitive regions in the human genome (39) RBMX2 belongs to the RNA-binding motif protein, and there are few studies at present (40). MESDC1 is considered the target gene of miRNA-508-5p. Overexpression of MESDC1 promotes the migration, invasion and proliferation of HepG2 cells in hepatocellular carcinoma. In bladder cancer, proliferation, migration and invasion were significantly inhibited by knockdown of MESDC1 in transfected bladder cell lines (41, 42). FUNDC2 is a mitochondrial outer membrane protein, and the novel FUNDC2/AKT/BCL-xL pathway prevents platelets from undergoing apoptotic stress (43). The PSMD6 gene encodes a member of the S10 family of protease subunits, and PSMD6 gene variation may be related to the therapeutic effect of diabetes (44). In glioblastoma, patients lacking XRCC6BP1 amplification have significantly longer survival (45). Tfip11 is the main homolog of the yeast splice cleavage factor ntr1 located in the nucleolus and Cajal body, which is very important for the 2’-O-methylation of U6. When TFIP11 is knocked down, fibrin and related snoRNA are less associated with U6 snRNA, altering U6 2’-O-methylation (46).

To assess the reliability of the risk prognostic model, we conducted external validation, and subgroup analysis. The AUC values of the ROC curves of 1-, 3-, and 5-year survival of the model were all greater than 0.7, which indicated that the signature composed of 11 SENP3-related genes had good performance in predicting the prognosis of TNBC patients.

Furthermore, by incorporating the risk score with customary clinical prognostic factors, including age and tumor stage, a high-precision predictive nomogram was constructed. The 1-, 3-, and 5-year OS probabilities of individual TNBC patients can be predicted based on conventional clinical prognostic parameters such as the risk score.

## Conclusion

Overall, we found that high levels of SENP3 were independent poor prognostic factors for TNBC patients. Knockdown of SENP3 inhibited the proliferation, invasion and migration of TNBC cells. In the future, we will carry out the relevant animal experiments and basic experimental research on SENP3, explore its regulatory mechanism, and provide new therapeutic targets for TNBC. In addition, we developed a clinically practical and easy-to-implement risk scoring model consisting of 11 SENP3-related genes. This model can serve as a potential biomarker to predict the prognosis of TNBC patients.

## Data availability statement

Publicly available datasets were analyzed in this study. This data can be found here: http://www.ncbi.nlm.nih.gov/geo/ http://www.cbioportal.org.

## Author contributions

XC, QW, and YL contributed to the conception and design. YZ, JZ, LY and SX contributed to the data acquisition and analysis. YZ, JZ, LY and LC contributed to the writing, review, and/or revision of the manuscript. LK, WL, KW and JX contributed to the study supervision. All authors read and approved the final manuscript. All authors contributed to the article and approved the submitted version.

## Supplementary material

Supplementary Figure 1 SENP3 expression in all subtypes of breast cancer **(A, B)** SENP3 expression in all subtypes of breast cancer based on **(A)** TCGA and GSE21653. *, *p* < 0.05; **, *p* < 0.01; ***, *p* < 0.001.

Supplementary Figure 2 Validation of the prognosis risk model The prognosis risk model was validated using GSE53752 database.
