# Correction: SENP3 promotes tumor progression and is a novel prognostic biomarker in triple-negative breast cancer

**DOI:** 10.3389/fonc.2026.1857283

**Published:** 2026-05-12

**Authors:** Youzhi Zhu, Jiasheng Zhang, Liangfei Yu, Sunwang Xu, Ling Chen, Kunlin Wu, Lingjun Kong, Wei Lin, Jiajie Xue, Qingshui Wang, Yao Lin, Xiangjin Chen

**Affiliations:** 1 Department of Thyroid and Breast Surgery, The First Affiliated Hospital of Fujian Medical University, Fuzhou, China; 2 Department of Breast Surgery, the First Hospital of Fuzhou, Fuzhou, China; 3 Key Laboratory of OptoElectronic Science and Technology for Medicine of Ministry of Education, College of Life Sciences, Fujian Normal University, Fuzhou, China; 4 Central Laboratory at The Second Affiliated Hospital of Fujian Traditional Chinese Medical University, Innovation and Transformation Center, Fujian University of Traditional Chinese Medicine, Fuzhou, China

**Keywords:** TNBC, SENP family, SENP3, WGCNA, prognosis

There was a mistake in **Figure 5D** as published. The incorrect images were inadvertently placed into the final figure, resulting in the misapplication of both the SCR and SENP3 sh-RNA2 panels. The corrected **Figure 5D** appears below.

**Figure 5:**
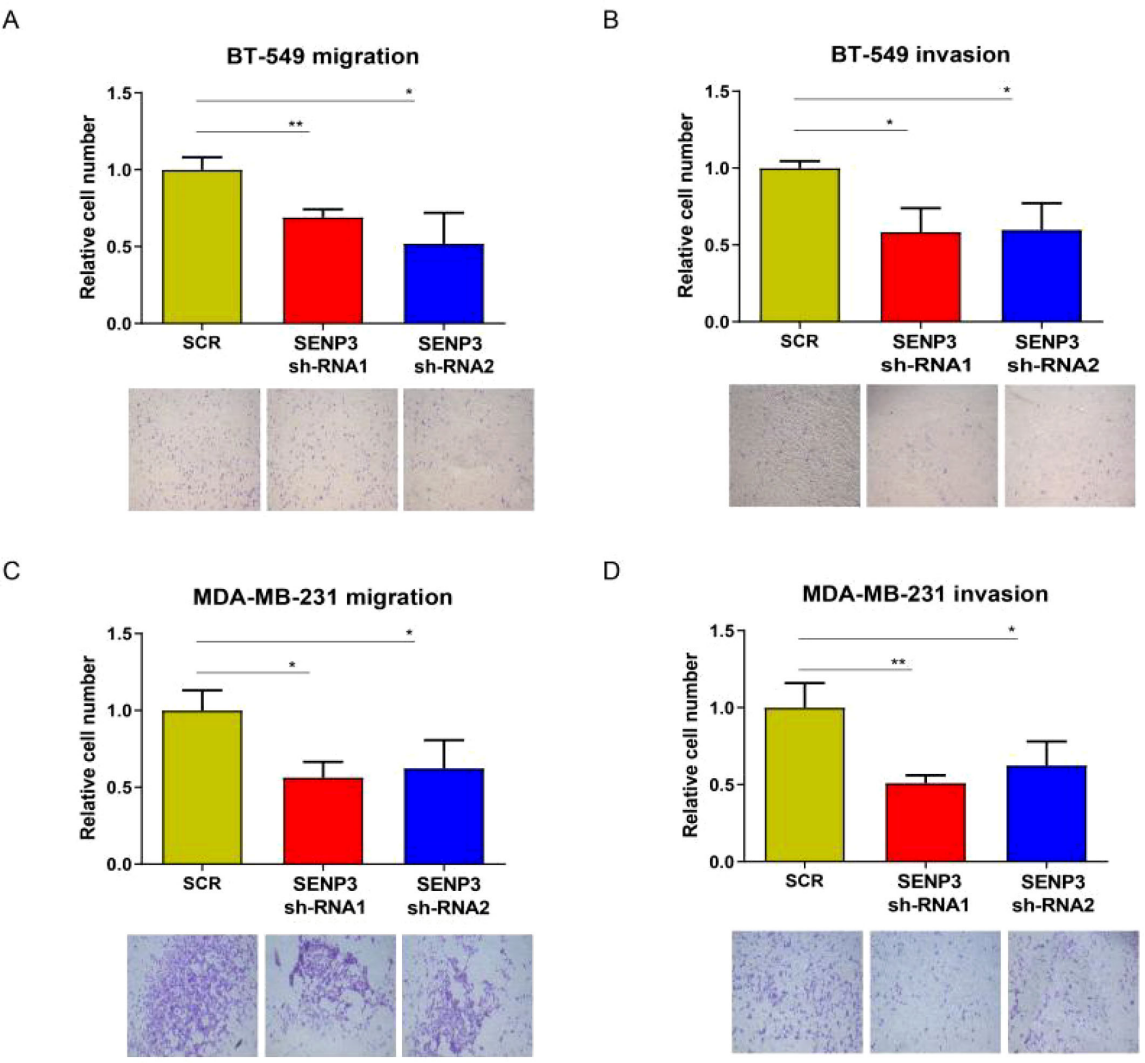
Knockdown of SNEP3 inhibits TNBC cell invasion and migration. **(A, B)** Decreased expression of SENP3 inhibited BT-549 cell migration. **(A)** and invasion. **(B)** Decreased expression of SENP3 inhibited MDA-MB-231 cell migration **(C)** and migration **(D)**. *, p < 0.05; **, p < 0.01.

The original version of this article has been updated.

