# Supplementary figures and images for: SENP3 promotes tumor progression and is a novel prognostic biomarker in triple-negative breast cancer
Source: Front Oncol. 2023 Jan 9;12:972969. doi: 10.3389/fonc.2022.972969 (PMC9868814; doi:10.3389/fonc.2022.972969)

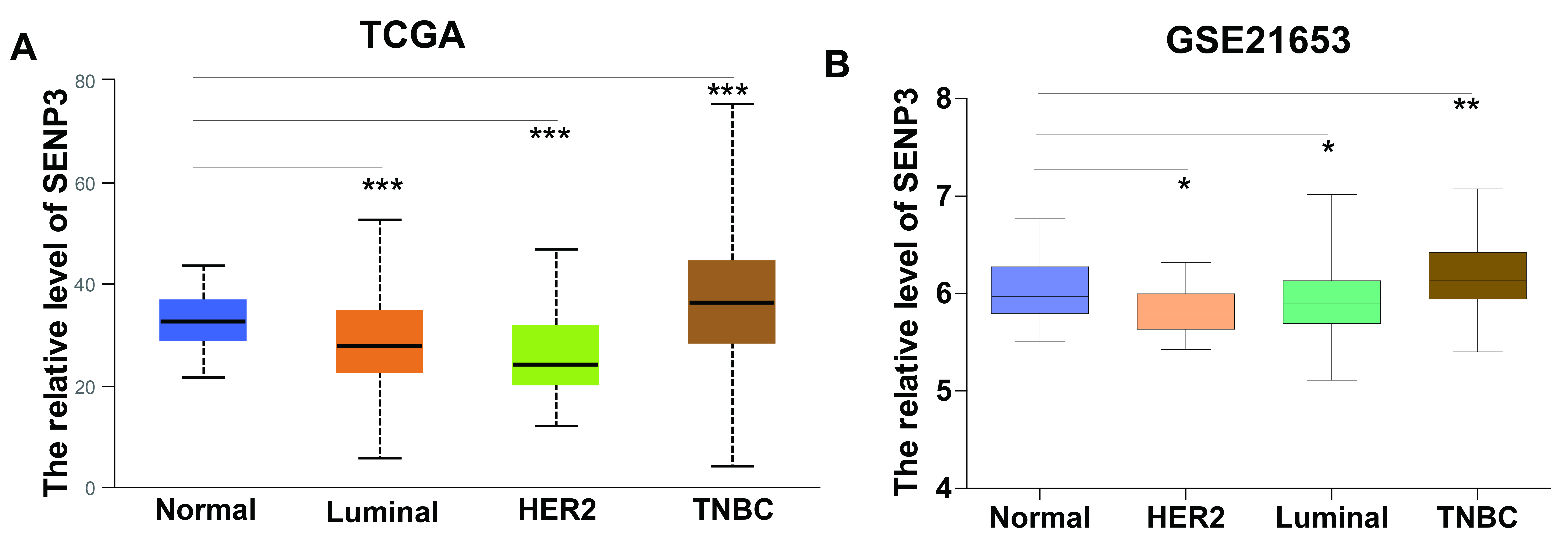

Supplement: Supplementary Figure 1 — SENP3 expression in all subtypes of breast cancer (A, B) SENP3 expression in all subtypes of breast cancer based on (A) TCGA and GSE21653. *, p < 0.05; **, p < 0.01; ***, p < 0.001. [file Image1.jpeg]

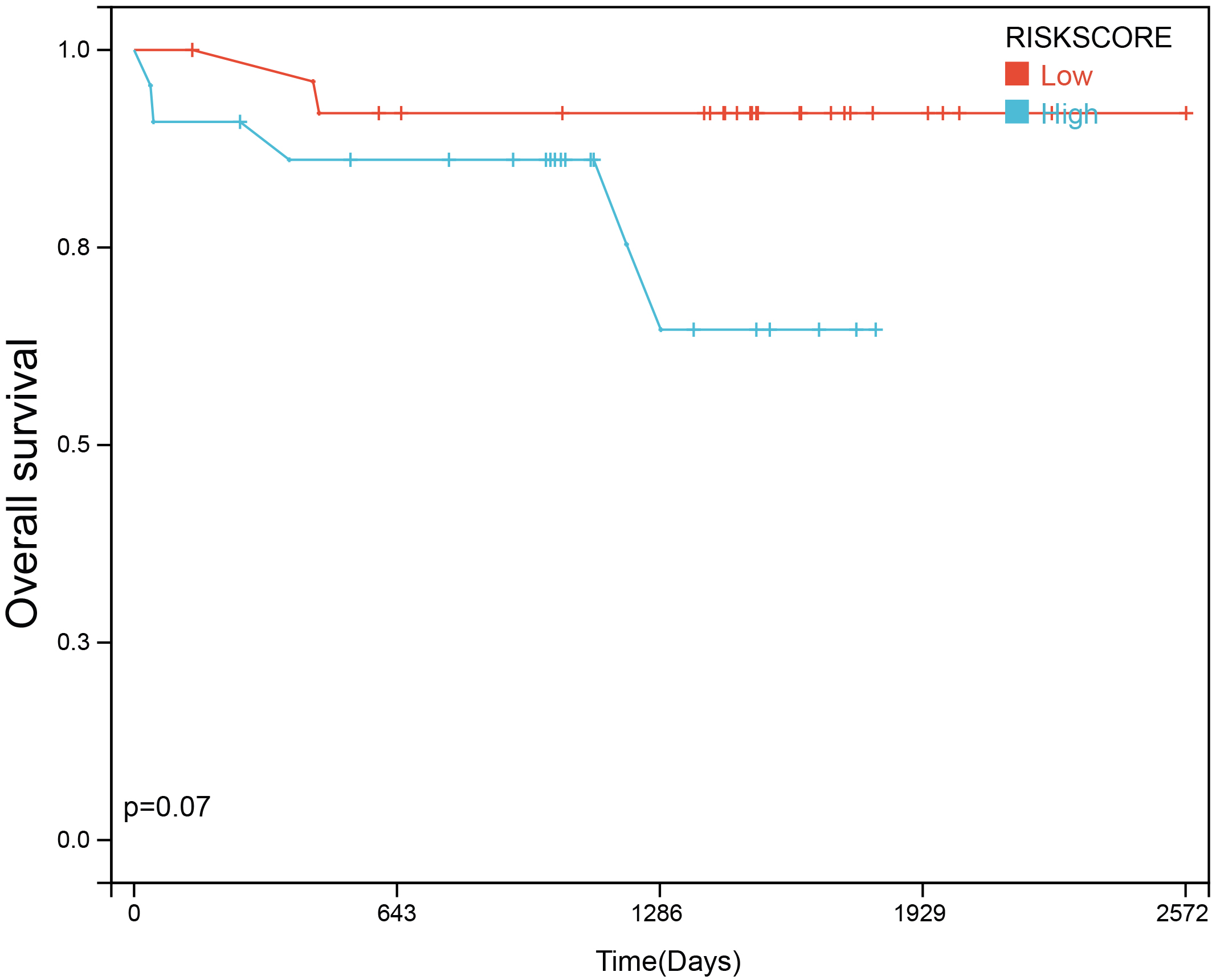

Supplement: Supplementary Figure 2 — Validation of the prognosis risk model The prognosis risk model was validated using GSE53752 database. [file Image2.jpeg]
